# Prognostic relevance of elevated plasma osmolality on admission in acute decompensated heart failure with preserved ejection fraction: insights from PURSUIT-HFpEF registry

**DOI:** 10.1186/s12872-021-02098-z

**Published:** 2021-06-07

**Authors:** Akito Nakagawa, Yoshio Yasumura, Chikako Yoshida, Takahiro Okumura, Jun Tateishi, Junichi Yoshida, Shunsuke Tamaki, Masamichi Yano, Takaharu Hayashi, Yusuke Nakagawa, Takahisa Yamada, Daisaku Nakatani, Shungo Hikoso, Yasushi Sakata, Osaka CardioVascular Conference (OCVC) Heart Failure Investigators

**Affiliations:** 1 Division of Cardiology, Amagasaki Chuo Hospital, 1-12-1 Shioe, Amagasaki, Hyogo 661-0976 Japan; 2 Department of Medical Informatics, Osaka University Graduate School of Medicine, 2-2 Yamadaoka, Suita, 565-0871 Japan; 3 Division of Cardiology, Osaka General Medical Center, 3-1-56 Mandaihigashi, Sumiyoshi-ku, Osaka 558-8558 Japan; 4 Division of Cardiology, Osaka Rosai Hospital, 3-1179 Nagasonecho, Kita-ku, Sakai 591-8025 Japan; 5 Cardiovascular Division, Osaka Police Hospital, 10-31 Kitayamacho, Tennojiku, Osaka 543-0035 Japan; 6 Division of Cardiology, Kawanishi City Hospital, 5-21-1, Kawanishi, Hyogo 666-0195 Japan; 7 Department of Cardiovascular Medicine, Osaka University Graduate School of Medicine, 2-2 Yamadaoka, Suita, 565-0871 Japan

**Keywords:** HFpEF, Plasma osmolality, Prognosis

## Abstract

**Background:**

Complicated pathophysiology makes it difficult to identify the prognosis of heart failure with preserved ejection fraction (HFpEF). While plasma osmolality has been reported to have prognostic importance, mainly in heart failure with reduced ejection fraction (HFrEF), its prognostic meaning for HFpEF has not been elucidated.

**Methods:**

We prospectively studied 960 patients in PURSUIT-HFpEF, a multicenter observational study of acute decompensated HFpEF inpatients. We divided patients into three groups according to the quantile values of plasma osmolality on admission. During a follow-up averaging 366 days, we examined the primary composite endpoint of cardiac mortality or heart failure re-admission using Kaplan–Meier curve analysis and Cox proportional hazard testing.

**Results:**

216 (22.5%) patients reached the primary endpoint. Kaplan–Meier curve analysis revealed that the highest quantile of plasma osmolality on admission (higher than 300.3 mOsm/kg) was significantly associated with adverse outcomes (Log-rank *P* = 0.0095). Univariable analysis in the Cox proportional hazard model also revealed significantly higher rates of adverse outcomes in the higher plasma osmolality on admission (hazard ratio [HR] 7.29; 95% confidence interval [CI] 2.25–23.92, *P* = 0.0009). Multivariable analysis in the Cox proportional hazard model also showed that higher plasma osmolality on admission was significantly associated with adverse outcomes (HR 5.47; 95% CI 1.46–21.56, *P* = 0.0113) independently from other confounding factors such as age, gender, comorbid of atrial fibrillation, hypertension history, diabetes, anemia, malnutrition, E/e′, and N-terminal pro-B-type natriuretic peptide elevation.

**Conclusions:**

Higher plasma osmolality on admission was prognostically important for acute decompensated HFpEF inpatients.

**Supplementary Information:**

The online version contains supplementary material available at 10.1186/s12872-021-02098-z.

## Introduction

There are many common problems in heart failure (HF) that are linked to hospitalization and mortality [1]. Heart failure with preserved ejection fraction (HFpEF) accounts for approximately half of all HF cases, and this rate is increasing [2]. Because of their pathophysiological complexity [3], the precise mechanisms involved in HFpEF with a poor prognosis are not fully understood.

Plasma osmolality is easily estimated with a blood sample as [4]:

1 $$2 \times \left[ {Serum\;sodium} \right] + \left[ {blood\;urea\;nitrogen} \right]/2.8 + \left[ {glucose} \right]/18$$

Although the components of the formula, namely sodium [5], blood urea nitrogen [6], serum glucose [7], and other parameters interacting with osmolality such as serum albumin [8] and renal function [9] have been proven to affect the prognosis of HF, little has been elucidated about the prognostic meaning of osmolality itself in acute decompensated HF (ADHF).

Plasma osmolality has been reported to be influenced by well-known prognostic factors such as arginine vasopressin (AVP), the renin–angiotensin–aldosterone system (RAAS), and natriuretic peptides [10–12], which suggests that osmolality itself could be also associated with the prognosis of HF. On one hand, Vaduganathan et al*.* reported that lower osmolality was associated with poor outcomes in HF with reduced ejection fraction (HFrEF) from a post hoc analysis of the EVEREST trial [13]. Kaya et al*.* also reported that low osmolality on admission correlated with a poor prognosis in HFrEF patients [14]. On the other hand, independent from left ventricular ejection fraction (LVEF), Arévalo-Lorido et al*.* reported higher osmolality in ADHF patients could predict worse outcomes accompanied by higher comorbidities through the National Registry of Heart Failure (RICA) [15].

Based on these previous reports, the aim of this study was to investigate further the prognostic meaning of plasma osmolality, particularly in acute decompensated HFpEF patients.

## Methods

### The PURSUIT-HFpEF registry

This prospective, multicenter, observational cohort study was performed in 1008 consecutive hospitalized HFpEF patients. Details of the PURSUIT-HFpEF (The **P**rospective m**U**lticente**R** ob**S**ervational st**U**dy of pat**I**en**T**s with **H**eart **F**ailure with **p**reserved **E**jection **F**raction) registry have been described previously [16]. Briefly, in collaboration with 31 hospitals in Japan, this large-scale registry aimed to collect and record a comprehensive range of clinical data to define the pathophysiology and prognostic factors of HFpEF patients. Inclusion criteria were acute decompensated HFpEF diagnosed by the Framingham criteria for HF and the following: 1) LVEF ≥ 50% and 2) N-terminal pro-B-type natriuretic peptide (NT-proBNP) ≥ 400 ng/L or brain natriuretic peptide (BNP) ≥ 100 ng/L on admission. Major exclusion criteria were age < 20 years, severe valvular diseases, acute coronary syndrome on admission, life expectancy of < 6 months due to prognosis of non-cardiac diseases, and previous heart transplantation. The anonymized data were transferred to the data center of Osaka University Hospital for analysis via data capturing system connected with electronic medical records [17]. Written informed consent was received from each participating patient. This study, including the procedure for enrollment, conformed to the principles of the Declaration of Helsinki and was approved by the institutional review board of each participating facility, including the official institutional review board committee of Osaka University Hospital (approved on February 24, 2016). It was registered under the Japanese UMIN Clinical Trials Registration (UMIN000021831).

### Study population

A total of 1024 inpatients with HFpEF were registered from June 2016 to February 2020. Of all the participants, 16 (1.6%) patients died in hospital. We should unfortunately exclude additional 48 patients due to missing of plasma osmolality on admission (missing of serum sodium; 1, blood urea nitrogen; 2, and glucose; 46). We finally analyzed remaining 960 (93.8%) patients discharged alive whose plasma osmolality was calculated on admission.

### Plasma osmolality, nutrition status, plasma volume estimation and echocardiographic measurements

Plasma osmolality was estimated [4] with a blood sample (Eq. 1). Nutrition status was estimated with the Geriatric Nutritional Risk Index (GNRI), which was calculated using serum albumin and body mass index as described previously [18]. Systemic plasma volume was estimated with plasma volume status (PVS) using hematocrit and body weight as described previously [19]. Comprehensive echocardiographic examinations were performed by trained cardiac sonographers according to the American Society of Echocardiography guidelines [20]. LVEF was calculated with the biplane Simpson’s method using apical two- and four-chamber views.

### Follow-up and endpoints

The primary endpoint of the present study was a composite of cardiac mortality or re-admission for HF during the follow-up period. The secondary endpoints were defined as each event of cardiac mortality and HF re-admission. The duration of the follow-up period was calculated from the day of discharge until an endpoint, or at the time of the last patient contact (including teleconferencing).

### Statistical analysis

Data are presented as median and interquartile range of 25–75% for continuous variables and frequency/percentage for categorical variables. Continuous variables were compared using Kruskal–Wallis test (and Steel–Dwass test for between each groups) and categorical variables were compared using Fisher’s exact test (with Bonferroni adjustment for between each groups). The distributions of plasma osmolality on admission and at discharge were compared with F-test. The correlation of plasma osmolality with sodium, urea nitrogen, glucose, and estimated glomerular filtration rate (GFR) were analyzed with linear regression models. The clinical endpoint was assessed with the Kaplan–Meier curve analysis and compared with the log-rank test. Univariable Cox proportional hazards regression models were used to calculate hazard ratios (HR) and 95% confidence intervals (CIs) for each endpoint. Multivariable Cox regression tests for plasma osmolality of our interest were performed using covariates of clinical importance as follows: age, gender, hypertension history, diabetes mellitus, hematocrit, GNRI, E/e’, and log-transformed NT-proBNP with and without estimated GFR. All statistical tests were 2-sided and *P* < 0.05 was regarded as statistically significant. Statistical analyses were performed using JMP® Pro 13.2.1, (SAS Institute Inc., Chicago IL, USA) or EZR version 1.51 (Saitama Medical Center, Jichi Medical University, Saitama, Japan).

## Results

### Characteristics of the study population

**Fig. 1:**
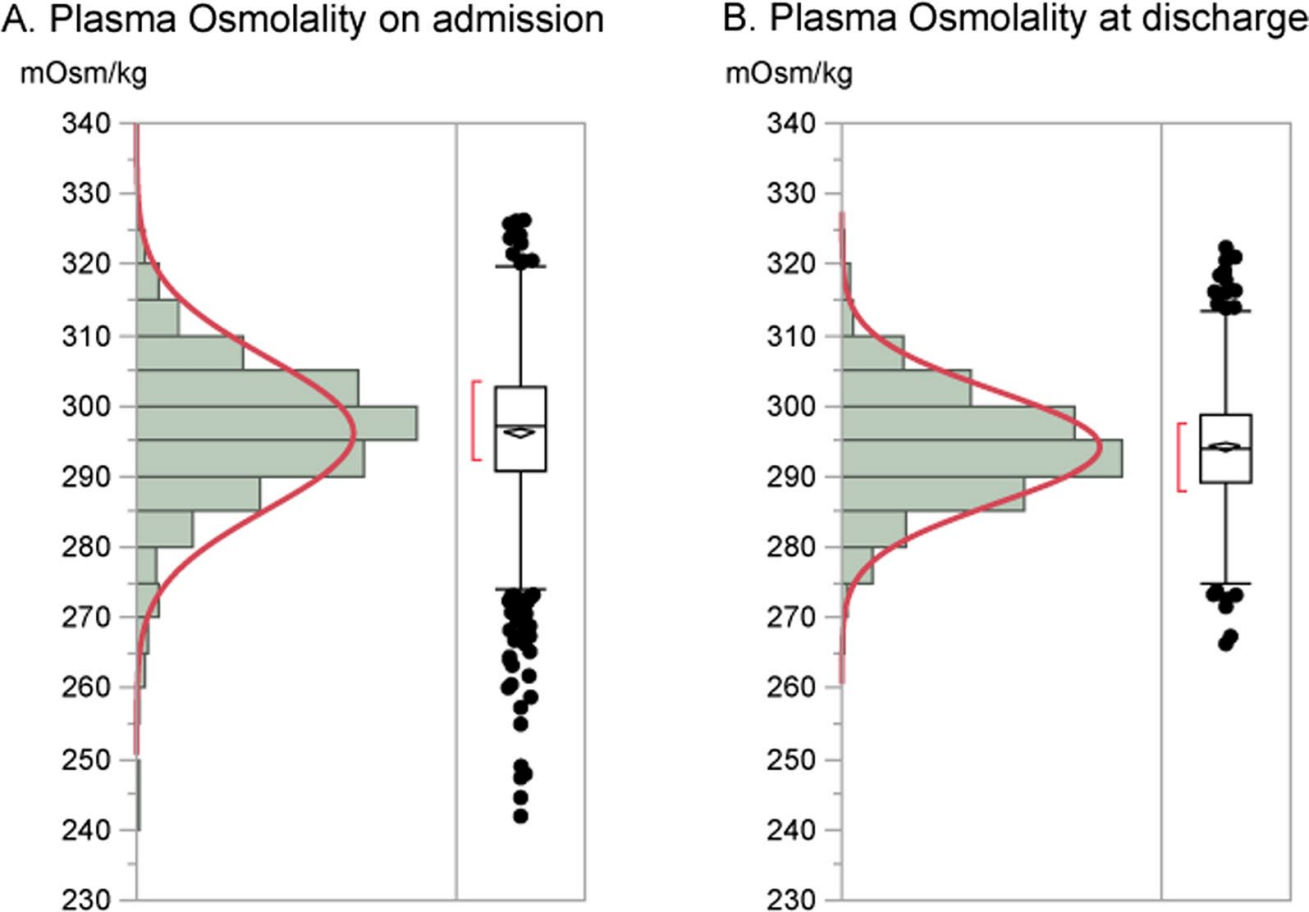
The distributions of plasma osmolality. Distributions of plasma osmolality on admission (**A**) and at discharge (**B**). F-test revealed that the distributions were significantly different between on admission and at discharge (*P* < 0.0001)

Distributions of plasma osmolality on admission and at discharge are shown in Fig. 1. Compared with the distribution at discharge, that on admission was significantly wide and shifted to higher levels (*P* < 0.0001, F-test). While the normal osmolality range is known to be 275–295 mOsm/kg [21], the median on admission was 297 mOsm/kg. Because plasma osmolality estimation consists of sodium, urea nitrogen, and glucose, osmolality had strong linear correlation with sodium (*r* = 0.797, *P* < 0.0001), and had mild correlation with urea nitrogen (*r* = 0.475, *P* < 0.0001) and with glucose (*r* = 0.221, *P* = 0.0001) (Additional file 1: Figure S1A, S1B, S1C).

Demographic and clinical characteristics of the 960 patients are summarized in the left column of Tables 1 and 2. The study population had a median age of 83 years; 55% were female. Hypertension (85%) was the most prevalent comorbidity followed by atrial fibrillation, dyslipidemia, and chronic kidney disease (46%, 41%, and 40%, respectively). The medians of NT-proBNP and estimated GFR were 3,250 ng/L and 45 mL/min/1.73 m^2^ on admission, respectively. In the first-step treatment, more than half of all patients were treated with a bolus injection of diuretics (57%); non-invasive positive pressure ventilation was used in 13%. The most frequent prescription at discharge was a loop diuretic (79%), which was the most increased treatment during hospitalization.

**Table 1:** Baseline characteristics and data on admission divided by plasma osmolality

	All patients (n = 960)	Q1 (n = 318) Osm < 293.2	Q2 (n = 322) 293.2 ≤ Osm < 300.3	Q3 (n = 320) 300.3 ≤ Osm	*P*-value
Age, years	83 (77–87)	83 (77–87)	83 (77–87)	83 (77–87)	0.9761
Female	524 (55)	178 (56)	179 (56)	167 (52)	0.5709
Prior HF hospitalization	244 (26)	63 (20)‡	84 (26)	97 (31)*	0.0089
*Comorbidities*					
Hypertension	809 (85)	254 (80)‡	272 (85)	183 (89)*	0.0112
Diabetes	314 (33)	87 (28)‡	93 (29)‡	134 (42)*,†	< 0.0001
Dyslipidemia	393 (41)	111 (35)‡	128 (40)	154 (48)*	0.0036
COPD	73 (8)	25 (8)	21 (7)	27 (9)	0.6247
CKD	384 (40)	97 (31)‡	118 (37)‡	169 (53)*,†	< 0.0001
Malignancy	112 (12)	36 (12)	33 (10)	43 (14)	0.4165
*General condition on admission*					
BMI, kg/m^2^	23.8 (21.0–26.9)	23.2 (20.6–26.5)‡	23.7 (20.9–26.8)	24.6 (21.9–27.7)*	0.0022
SBP, mmHg	147 (128–170)	146 (129–166)	149 (127–167)	149 (128–175)	0.3444
DBP, mmHg	80 (66–93)	82 (69–92)	80 (67–94)	76 (64–93)	0.2598
Heart rate	82 (67–100)	82 (68–102)	82 (68–99)	82 (65–100)	0.7596
AF	444 (46)	153 (48)	154 (48)	137 (43)	0.3187
GNRI	98 (90–106)	96 (89–103)‡	98 (90–106)	100 (92–107)*	0.0110
*Laboratory examination on admission*					
Hemoglobin, g/dL	11.1 (9.8–12.5)	11.5 (10.1–12.7)‡	11.4 (10.1–12.7)‡	10.7 (9.4–12.3)*,†	< 0.0001
Hematocrit, %	34 (30–38)	35 (31–38)‡	35 (31–38)‡	33 (29–38)*,†	0.0033
Serum total protein, g/dL	6.7 (6.3–7.1)	6.7 (6.3–7.2)	6.7 (6.3–7.2)	6.7 (6.2–7.1)	0.3454
Serum albumin, g/dL	3.5 (3.2–3.8)	3.5 (3.1–3.8)	3.5 (3.2–3.9)	3.5 (3.1–3.8)	0.3340
BUN, mg/dL	22 (16–32)	18 (14–24)†,‡	21 (15–27)*,‡	31 (23–43)*,†	< 0.0001
Creatinine, mg/dL	1.1 (0.8–1.5)	1.0 (0.7–1.2)†,‡	1.0 (0.8–1.3)*,‡	1.4 (0.9–2.0)*,†	< 0.0001
eGFR, mL/min/1.73m^2^	45 (30–58)	51 (38–65)†,‡	45 (33–59)*,‡	33 (21–50)*,†	< 0.0001
Serum sodium, mEq/L	140 (137–142)	137 (134–138)†,‡	141 (139–142)*,‡	142 (140–144)*,†	< 0.0001
Serum potassium, mEq/L	4.1 (3.7–4.5)	4.2 (3.8–4.5)†	4.0 (3.7–4.4)*,‡	4.2 (3.7–4.6)†	0.0084
Serum chloride, mEq/L	105 (101–108)	101 (98–105)†,‡	105 (103–108)*,‡	107 (104–110)*,†	< 0.0001
NT-proBNP, ng/L	3250 (1718–6430)	2950 (1637–5281‡	2820 (1580–5292)‡	4805 (2108–10,010)*,†	< 0.0001
CRP, mg/dL	0.53 (0.19–1.94)	0.64 (0.21–2.43)	0.46 (0.18–1.47)	0.53 (0.20–2.00)	0.0734
Glucose, mg/dL	122 (103–161)	118 (101–146)‡	117 (102–147)‡	133 (112–194)*,†	< 0.0001
PVS, %	8.7 (− 0.4–16.8)	8.3 (− 0.04–17.4)	7.4 (− 1.1–15.6)	9.8 (0.6–17.9)	0.3156
Plasma osmolality, mOsm/kg	297 (291–303)	288 (283–291)†,‡	297 (295–299)*,‡	305 (303–309)*,†	< 0.0001
*Echocardiographic variables on admission*					
LVDd, mm	46 (42–51)	46 (41–49)‡	46 (41–51)	47 (43–51)*	0.0066
LVEF, %	60 (56–65)	60 (56–67)	61 (56–65)	60 (55–65)	0.8524
LAD, mm	44 (40–50)	44 (39–49)	45 (40–51)	45 (39–50)	0.1973
E/A	1.0 (0.7–1.5)	1.1 (0.7–1.5)	1.1 (0.7–1.7)	0.9 (0.7–1.5)	0.4369
E/e′	16 (12–21)	16 (12–20)‡	16 (12–21)	17 (13–22)*	0.0311
TRPG, mmHg	36 (28–45)	36 (28–45)	36 (29–45)	36 (29–45)	0.9959
IVC max, mm	19 (15–22)	18 (15–22)	18 (15–22)‡	19 (16–22)†	0.0548
IVC collapsibility	0.44 (0.28–0.57)	0.38 (0.23–0.55)†	0.48 (0.30–0.59)*	0.44 (0.29–0.56)	0.0009
*Acute phase treatment*					
NIPPV usage	121 (13)	36 (11)‡	26 (8)‡	59 (19)*,†	0.0003
intubation	16 (1.7)	3 (0.9)	2 (0.6)‡	11 (3)†	0.0094
DOA (continuous injection)	1 (0.1)	0 (0)	0 (0)	1 (0.3)	0.3663
DOB (continuous injection)	17 (1.7)	2 (0.6)	7 (2)	8 (3)	0.1591
NAD (continuous injection)	10 (1.0)	3 (0.9)	3 (0.9)	4 (1.3)	0.9018
PDE3I (continuous injection)	3 (0.3)	0 (0)	2 (0.6)	1 (0.3)	0.3717
Carperitide (continuous injection)	207 (22)	54 (17)‡	66 (21)	87 (27)*	0.0063
nitrates (continuous injection)	264 (28)	85 (27)	80 (25)	99 (31)	0.1989
Calcium channel blocker (continuous injection)	77 (8)	18 (6)‡	23 (7)	36 (11)*	0.0255
Nicorandil (continuous injection)	6 (0.6)	1 (0.3)	3 (0.9)	2 (0.6)	0.6125
Diuretics (continuous injection)	310 (32)	103 (32)	100 (31)	107 (34)	0.7970
Diuretics (bolus injection)	549 (57)	176 (55)	178 (55)	195 (61)	0.2296
*Prescription before admission*					
Antiplatelet	292 (30)	91 (29)	95 (30)	106 (33)	0.4225
ACE inhibitor or ARB	481 (50)	149 (47)	156 (48)	176 (55)	0.0924
Calcium channel blocker	489 (51)	148 (47)	162 (50)	179 (56)	0.0575
β-blocker	444 (46)	136 (43)	155 (48)	153 (48)	0.3031
Loop diuretics	483 (50)	133 (42)‡	165 (51)	185 (58)*	0.0003
Thiazide	72 (8)	33 (10)†	13 (4)*	26 (8)	0.0085
Tolvaptan	52 (5)	14 (4)	22 (7)	16 (5)	0.3667
Aldosterone antagonist	204 (21)	72 (23)	68 (21)	64 (20)	0.7153
SGLT2 inhibitor	15 (1.6)	6 (1.9)	2 (0.6)	7 (2)	0.2363
Anticoagulant	424 (44)	154 (48)	145 (45)	125 (39)	0.0545

ACE, angiotensin-converting enzyme; AF, atrial fibrillation; ARB, angiotensin receptor blocker; BMI, body mass index; BUN, blood urea nitrogen; CKD, chronic kidney disease; COPD, chronic obstructive pulmonary disease; CRP,C-reactive protein; DBP, diastolic blood pressure; DOA, dopamine; DOB, dobutamine; eGFR, estimated glomerular filtration rate; GNRI, Geriatric Nutritional Risk Index; HF, heart failure; IVC, inferior vena cava; LAD, left atrial dimension; LVDd, left ventricular end-diastolic diameter; LVEF, left ventricular ejection fraction; NAD, noradrenaline; NIPPV, noninvasive positive pressure ventilation; NT-proBNP, N-terminal pro-B-type natriuretic peptide; Osm, plasma osmolality (mOsm/kg); PCI, percutaneous catheter intervention; PDE3I,phosphodiesterase-3 inhibitor; PVS, plasma volume status; SBP, systolic blood pressure; SGLT2, sodium glucose cotransporter 2; TRPG, tricuspid regurgitation pressure gradient

Values are given as median (IQR) or n (%)

Statistical comparisons were performed using Kruskal Wallis test or Fisher’s exact test. Statistical significances between each group (*P* < 0.05) using Steel–Dwass test for continuous variables and Fisher’s exact test with Bonferroni adjustment for categorical variables are shown as following: significance in versus Q1*, versus Q2†, and versus Q3‡

**Table 2:** Clinical and study characteristics at discharge divided by plasma osmolality on admission

General condition at discharge	All patients (n = 960)	Q1 (n = 318) Osm < 293.2	Q2 (n = 322) 293.2 ≤ Osm < 300.3	Q3 (n = 320) 300.3 ≤ Osm	*P*-value
BMI, kg/m^2^	21.4 (18.9–24.2)	21.1 (18.4–23.8)‡	21.2 (18.7–24.3)	21.9 (19.4–24.6)*	0.0150
SBP, mmHg	118 (106–131)	117 (106–128)‡	118 (106–130)	122 (107–134)*	0.0106
DBP, mmHg	65 (58–73)	65 (58–73)	66 (58–74)	65 (57–73)	0.7041
Heart rate	70 (61–80)	70 (63–80)	70 (61–80)	70 (60–78)	0.5914
AF	365 (38)	124 (39)	131 (41)	110 (34)	0.2242
GNRI	92 (85–99)	91 (84–97)	94 (85–101)	92 (85–99)	0.1277
6MWD, m	260 (155–340)	240 (150–333)	270 (156–352)	260 (160–338)	0.5728
*NYHA classification*					0.6460
NYHA I	340 (36)	111 (36)	106 (33)	123 (39)	
NYHA II	538 (57)	173 (55)	193 (60)	172 (54)	
NYHA III	67 (7)	26 (8)	20 (6)	21 (7)	
NYHA IV	4 (0.4)	2 (0.6)	1 (0.3)	1 (0.3)	
*Laboratory examination at discharge*					
Hemoglobin, g/dL	11.3 (10.1–12.7)	11.5 (10.3–12.7)‡	11.6 (10.4–13.1)‡	10.8 (9.5–12.2)*,†	< 0.0001
Hematocrit, %	34 (31–39)	35 (32–38)‡	35 (32–39)‡	33 (30–37)*,†	< 0.0001
Serum total protein, g/dL	6.6 (6.2–7.1)	6.8 (6.3–7.2)‡	6.8 (6.3–7.2)‡	6.5 (6.1–7.0)*,†	0.0009
Serum albumin, g/dL	3.4 (3.1–3.7)	3.4 (3.1–3.7)	3.4 (3.2–3.8)‡	3.3 (3.1–3.6)†	0.0104
BUN, mg/dL	25 (18–34)	22 (16–28)†,‡	25 (18–33)*,‡	29 (21–42)*,†	< 0.0001
Creatinine, μmol/L	1.1 (0.9–1.5)	1.0 (0.8–1.2)†,‡	1.1 (0.9–1.5)*,‡	1.3 (1.0–2.1)*,†	< 0.0001
eGFR, mL/min/1.73m^2^	42 (30–55)	50 (37–60)†,‡	42 (32–54)*,‡	33 (21–49)*,†	< 0.0001
Serum sodium, mEq/L	139 (137–141)	138 (135–140)†,‡	140 (138–141)*,‡	140 (138–142)*	< 0.0001
Serum potassium, mEq/L	4.3 (3.9–4.6)	4.3 (3.9–4.6)	4.3 (4.0–4.6)	4.3 (3.9–4.6)	0.8271
Serum chloride, mEq/L	103 (100–106)	102 (99–105†,‡	103 (100–105)*,‡	104 (101–107)*,†	< 0.0001
NT–proBNP, ng/L	1112 (478–2550)	993 (497–2190)‡	952 (439–2025)‡	1437 (510–3770)*,†	0.0010
CRP, mg/dL	0.29 (0.11–0.90)	0.34 (0.11–1.01)	0.28 (0.11–0.77)	0.26 (0.11–0.93)	0.4804
Glucose, mg/dL	98 (88–117)	97 (87–114)	98 (88–117)	101 (89–120)	0.3746
PVS, %	11.5 (1.9–19.6)	9.9 (1.9–20.0)	9.6 (0.9–17.9)‡	13.4 (3.1–21.2)†	0.0411
Plasma osmolality, mOsm/kg	294 (289–299)	290 (286–295)†,‡	294 (290–299)*,‡	297 (293–302)*,†	< 0.0001
*Prescription at discharge*					
Antiplatelet	278 (29)	82 (26)	93 (29)	103 (32)	0.1946
ACE inhibitor or ARB	510 (53)	157 (49)	168 (52)	185 (58)	0.0935
Calcium channel blocker	458 (48)	135 (42)‡	149 (46)	174 (55)*	0.0076
β-blocker	526 (55)	167 (53)	181 (56)	178 (56)	0.5896
Loop diuretics	754 (79)	243 (76)	254 (79)	257 (80)	0.4793
Thiazide	62 (6)	18 (6)	16 (5)	28 (9)	0.1165
Tolvaptan	156 (16)	39 (12)‡	54 (17)	63 (20)*	0.0377
Aldosterone antagonist	383 (40)	125 (39)	141 (44)	117 (37)	0.1683
SGLT2 inhibitor	50 (5)	13 (4)	12 (4)	25 (8)	0.0356
Anticoagulant	571 (59)	206 (65)‡	198 (61)	167 (52)*	0.0035

6MWD, 6-min walk distance; ACE, angiotensin-converting enzyme; AF, atrial fibrillation; ARB, angiotensin receptor blocker; BMI, body mass index; BUN, blood urea nitrogen; CRP, C-reactive protein; DBP, diastolic blood pressure; eGFR, estimated glomerular filtration rate; GNRI, Geriatric Nutritional Risk Index; NT-proBNP, N-terminal pro-B-type natriuretic peptide; NYHA, New York heart failure functional class; Osm, plasma osmolality (mOsm/kg); PVS, plasma volume status; SBP, systolic blood pressure; SGLT2, sodium glucose cotransporter 2

Values are given as median (IQR) or n (%)

Statistical comparisons were performed using Kruskal Wallis test or Fisher’s exact test. Statistical significances between each group (*P* < 0.05) using Steel–Dwass test for continuous variables and Fisher’s exact test with Bonferroni adjustment for categorical variables are shown as following: significance in versus Q1*, versus Q2†, and versus Q3‡

According to the quantiles of plasma osmolality on admission (293.2 and 300.3 mOsm/kg), we divided patients into three groups. Background and general information on admission are described in the right column of Table 1. Among the components for the osmolality calculation (serum sodium, blood urea nitrogen, and glucose), serum sodium and blood urea nitrogen were significantly elevated in accordance with the elevation of plasma osmolality. In the higher osmolality groups, hypertension, diabetes mellitus, dyslipidemia and chronic kidney disease were prevalent. The higher osmolality groups showed renal dysfunction, and NT-proBNP of the highest quantile group (Q3) was significantly higher than those in other groups. Echocardiography on admission showed generally comparable between groups. In acute phase treatment, intravenous usage of carperitide was more frequent in the Q3 group. At discharge (right column of Table 2) higher osmolality groups on admission still had higher osmolality at discharge, and the medians plasma osmolality of Q2 and Q3 had decreased to just around the upper limit of normal. The higher osmolality groups also had significantly lower estimated GFR compared with lower osmolality groups.

### Plasma osmolality and prognosis

**Fig. 2:**
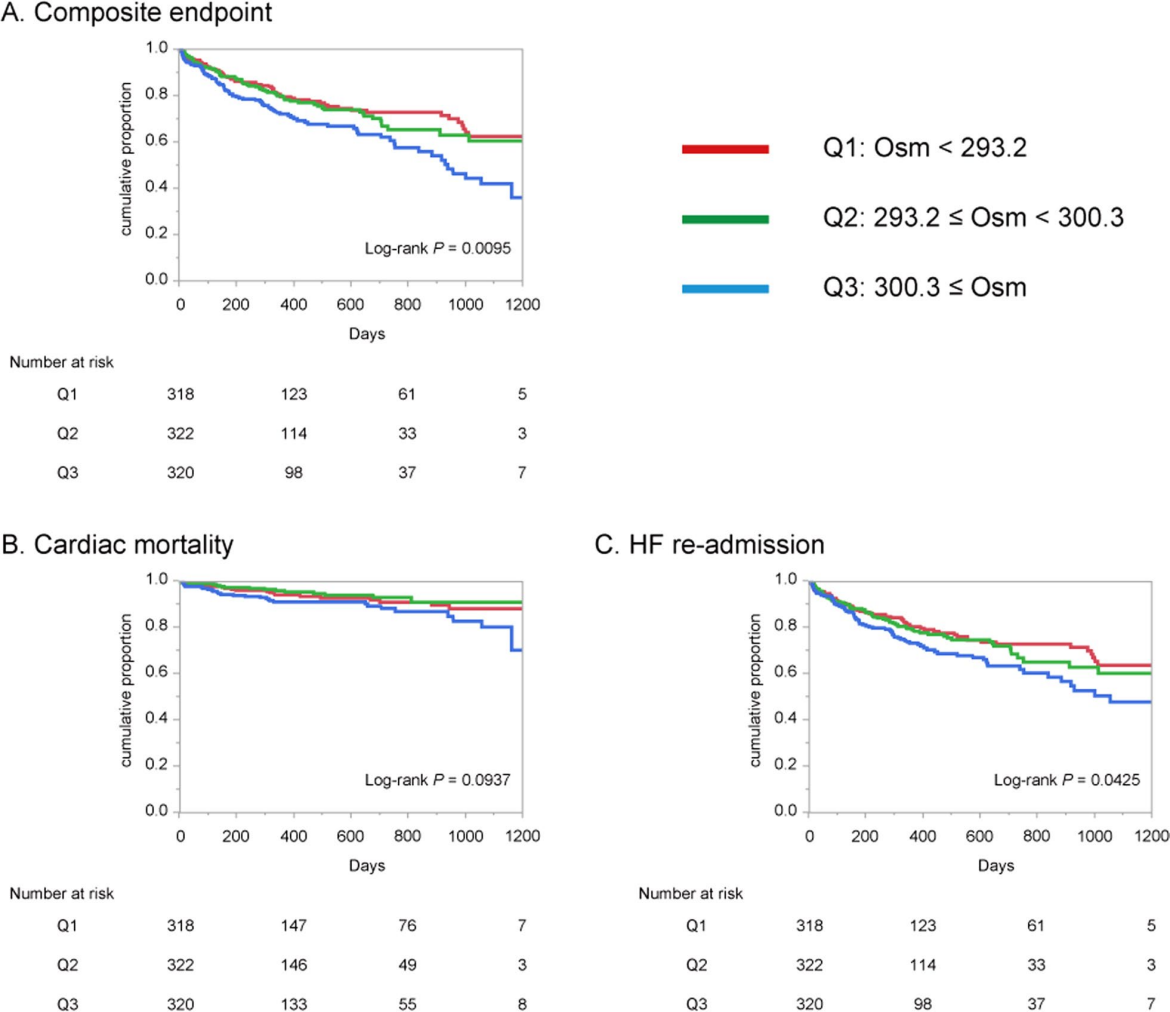
Kaplan–Meier survival curves stratified with quantiles of plasma osmolality on admission. Composite endpoint was defined as cardiac mortality or heart failure re-admission. Kaplan–Meier survival curves for composite endpoint (**A**), cardiac mortality (**B**) and heart failure re-admission (**C**). HF, heart failure; Osm, plasma osmolality (mOsm/kg) on admission

Among 960 patients, 216 patients (22.5%) suffered composite endpoint with a mean ± standard deviation (SD) follow-up of 366 ± 356 days. As far as the secondary endpoint, 62 patients (6.5%) reached cardiac mortality in 444 ± 378 days, 204 (21.3%) re-admitted for HF in 366 ± 356 days. Receiver operating curve analysis provided that the ideal cut-off value of plasma osmolality on admission for predicting the primary endpoint was 299 mOsm/kg (area under the curve; 0.563, sensitivity; 0.50, specificity; 0.62, *P* = 0.0046), which was almost equal to the median (297 mOsm/kg) of the whole cohort. The Kaplan–Meier curves revealed that higher plasma osmolality was significantly associated with the primary endpoint (Log-rank *P* = 0.0095) (Fig. 2). Univariable Cox regression tests revealed that the significance was observed particularly between the highest osmolality group (Q3) versus the lowest osmolality group (Q1) (HR 1.61; 95% CI 1.16–2.23, *P* = 0.0120) (Table 3). Regarding the secondary endpoint, in the Kaplan–Meier curve analyses, HF readmission was also significantly more frequent in the higher osmolality group (Log-rank *P* = 0.0425), which was not in case with cardiac mortality (Log-rank *P* = 0.0937) (Fig. 2). Through univariable Cox regression tests for clinically important parameters on admission, higher age, lower hematocrit, lower eGFR, higher NT-proBNP, higher E/e’, and higher plasma osmolality were associated with the primary endpoint (left column of Table 4). Whereas only the age was shown to be independently associated with the primary endpoint through multivariable Cox regression analysis (center column of Table 4), plasma osmolality was also found to be also independently associated when eGFR was avoided from the confounders (right column of Table 4). Because plasma osmolality showed mild linear correlation with eGFR (*r* =  − 0.379, *P* < 0.0001, Additional file 1: Figure S1D), it should be possible that plasma osmolality and eGFR had conflict in the multivariable analysis.

**Table 3:** Cox regression models for prognostic prediction, divided with the internal quantile ranges of plasma osmolality on admission

	Unadjusted HR (95% CI)	*P*-value
*Composite endpoint*		
Q2 versus Q1	1.13 (0.80–1.60)	1.0000
Q3 versus Q1	1.61 (1.16–2.23)	0.0120
Q3 versus Q2	1.42 (1.03–1.96)	0.0954
*Cardiac mortality*		
Q2 versus Q1	0.85 (0.42–1.66)	1.0000
Q3 versus Q1	1.59 (0.89–2.88)	0.3531
Q3 versus Q2	1.88 (1.01–3.61)	0.1338
*HF re-admission*		
Q2 versus Q1	1.12 (0.79–1.60)	1.0000
Q3 versus Q1	1.50 (1.08–2.11)	0.0504
Q3 versus Q2	1.34 (1.08–2.11)	0.2454

HF, heart failure; HR, hazard ratio; Q1, plasma osmolality on admission < 293.2 mOsm/kg; Q2, plasma osmolality on admission ≥ 293.2 and < 300.3 mOsm/kg; and Q3, plasma osmolality on admission ≥ 300.3 mOsm/kg

Cox proportional hazard models for composite endpoint, cardiac mortality and heart failure re-admission. Composite endpoint was defined as cardiac mortality or heart failure re-admission. *P*-value was corrected with Bonferroni adjustment

**Table 4:** Cox regression models for prognostic prediction of the primary endpoint

	Unadjusted HR (95% CI)	*P*-value	Adjusted HR (95% CI)	*P*-value	Adjusted HR (95% CI)	*P*-value
Age	5.86 (1.88–19.32)	0.0019	5.67 (1.40–24.73)	0.0143	5.98 (1.53–25.19)	0.0093
Female	1.20 (0.91–1.58)	0.1897	1.19 (0.85–1.68)	0.3120	1.18 (0.85–1.65)	0.3356
AF	0.96 (0.74–1.26)	0.7902	1.15 (0.82–1.62)	0.4163	1.11 (0.79–1.56)	0.5309
HT	0.94 (0.77–1.15)	0.5207	0.87 (0.54–1.48)	0.5952	0.89 (0.55–1.52)	0.6637
Diabetes	0.94 (0.80–1.10)	0.4475	1.18 (0.83–1.67)	0.3447	1.19 (0.84–1.68)	0.3242
hematocrit	0.28 (0.10–0.77)	0.0139	1.08 (0.33–3.41)	0.9028	0.84 (0.27–2.53)	0.7555
eGFR	0.15 (0.07–0.34)	< 0.0001	0.39 (0.12–1.24)	0.1110		
GNRI	0.40 (0.10–1.57)	0.1907	0.66 (0.19–2.36)	0.5257	0.80 (0.23–2.72)	0.7170
Log NT-proBNP	15.04 (1.93–60.70)	0.0139	1.73 (0.46–6.57)	0.4196	2.69 (0.80–8.96)	0.1077
E/e’	3.29 (1.25–7.84)	0.0168	2.07 (0.61–6.38)	0.2372	1.99 (0.59–6.09)	0.2607
Plasma Osmolality	7.29 (2.25–23.92)	0.0009	3.51 (0.89–17.35)	0.0730	5.47 (1.46–21.56)	0.0113
BUN	6.87 (3.36–13.52)	< 0.0001				
Serum sodium	1.18 (0.39–3.81)	0.7755				
Glucose	1.58 (0.46–4.66)	0.4447				

AF, atrial fibrillation; BUN, blood urea nitrogen; eGFR, estimated glomerular filtration rate; GNRI, Geriatric Nutritional Risk Index, HT, Hypertension; HR, hazard ratio; NT-proBNP, N-terminal pro-B-type natriuretic peptide

Composite endpoint was defined as cardiac mortality or heart failure re-admission

We further examined the event risk of a composite endpoint among the quantiles stratified by plasma osmolality at discharge. The Kaplan–Meier curve showed that the event risk was not associated with the osmolality at discharge in this cohort (Log-rank *P* = 0.1976, Additional file 1: Figure S2).

## Discussion

In this study, we showed that higher plasma osmolality on admission was significantly associated with the adverse outcomes for HFpEF patients. Although a few reports have also indicated that plasma osmolality had prognostic meaning for HF patients, their descriptions were so scattered that we were unable to reach a consensus on how to deal with this marker. Thus, our present finding in a prospective cohort that “higher plasma osmolality on admission” was associated with the adverse outcomes in “hospitalized decompensated HFpEF” patients is notable.

### Prognostic difference in plasma osmolality between HFpEF and HFrEF

Though a sub-analysis of the EVEREST trial for HFrEF patients, Vaduganathan *et.al* showed that normal osmolality at discharge was associated with improved outcomes [13]. Kaya *et.al* investigated clinical implication of plasma osmolality on admission for HFrEF patients [14]. They presented the third quartile of normo-to-hyperosmolality (mean of 293 mOsm/kg) as having the smallest adverse outcome rates, while the lowest quartile (mean of 280 mOsm/kg) showed the worst outcomes, followed by the highest quartile (mean of 301 mOsm/kg). According to these studies, plasma osmolality in the normal range seemed to be quite important for HFrEF patients. This finding should also be related to the particular prognostic importance of hyponatremia in HFrEF [22]. Contrary to these reports, Arévalo-Lorido *et.al* reported that the frequency of adverse outcomes increased in accordance with the increase in osmolality on admission in ADHF [15], similarly to our findings. Although their registry did not group subjects by LVEF, about 70% of the patients had LVEFs > 45%, indicating that HFrEF was underrepresented in that cohort. Taken our present findings together with those of Arévalo-Lorido *et.al*, we conclude that the elevation of plasma osmolality on admission raises the predictability of adverse outcomes in decompensated HFpEF patients.

### Cause of higher plasma osmolality in HFpEF patients

Different from HFrEF patients, higher plasma osmolality on admission was related to adverse outcomes in HFpEF patients. It should be noted that the plasma osmolality on admission in our HFpEF cohort (median of 297 mOsm/kg, Table 1) was generally higher than that of a previous reported HFrEF cohort (median of approximately 290 mOsm/kg) [14]. In an experimental study [12], excessive RAAS activation was proven to cause osmolality elevation in the acute phase of a rapid pacing HF model. RAAS activation could cause sodium reabsorption through modulation of the GFR, tubuloglomerular feedback, glomerulotubular balance, and distal tubular reabsorption [23], which could increase plasma osmolality. Relative hypovolemia in the higher osmolality groups compared with the lower osmolality groups was not likely to be the cause of RAAS activation because PVS was comparable between groups (Table 1). AVP is known to be an another cause of volume retention, and increased AVP activity causes a decrease in osmolality accompanied by hyponatremia in HFrEF patients [24]. In contrast, age-related attenuation of the AVP response [25] could be more common in elderly HFpEF patients than in younger HFrEF patients. Excessive RAAS activation compared to AVP activity might contribute to the higher plasma osmolality in HFpEF compared to HFrEF patients.

Regarding echocardiographic parameters reflecting hemodynamics, tricuspid regurgitation pressure gradient was not different at all between three groups, and inferior vena cava collapsibility was significantly different but did not coincide with the prognostic result, namely in which collapsibility was significantly impaired in the Q1 group (Table 1). Although E/e′ tended to be higher in the poor prognostic Q3 group, multivariable Cox regression models showed independent prognostic importance of plasma osmolality from E/e’ (Table 4). Considering these results, prognostic predictability based on the osmolality classification seemed to be unrelated to the hemodynamic observed in echocardiography. Despite these echocardiographic parameters, poor prognostic Q3 group showed frequent usage of intubation, non-invasive positive pressure ventilator, carperitide, and calcium channel blocker injection for initial treatment, frequent prior usage of loop diuretics, and higher NT-proBNP elevation on admission. The frequent usage of loop diuretics might directly affect the increase of plasma osmolality on admission [26]. These aspects ensured that Q3 group was in more decompensated hemodynamic status on admission paradoxically from the echocardiographic observations, and it seemed reasonable that those patients served poor outcomes.

### Prognostic implication of higher plasma osmolality on admission

We showed that higher plasma osmolality on admission was associated with poorer prognosis in HFpEF patients. The prognostic impact of the AVP system in HF has not been fully elucidated. Because of the short half-life of AVP, it is not practical to measure plasma AVP as a prognostic marker. In this point, copeptin has attracted attention owing to its creation from prepro-vasopressin at the same time as AVP and longer half-life [27]. Some reports have shown the prognostic implications of copeptin for HF [28, 29]. Although plasma osmolality on admission is not necessarily determined by the AVP system, there is a report consistent with our findings. Hage *et al* described the prognostic meaning of copeptin in a prospective HFpEF cohort (KaRen-study) and clarified that copeptin was elevated in HFpEF patients and had partial prognostic implications, which were blunted after adjustment for NT-proBNP [30]. The relevance of neurohormonal balance and pathophysiology in HFpEF should be further investigated.

### What are the clinical implications?

The following variables have reported as prognostic markers in the acute phase in HFpEF patients: TRPG [31], lung congestion observed as B-lines (‘comets’) on lung ultrasound [32], soluble suppression of tumorigenesis-2 with NT-proBNP [33] and cystatin C [34]. In addition to these factors, our findings showed that higher plasma osmolality also has important prognostic implications in the acute phase of HFpEF.

The higher osmolality groups presented even higher plasma osmolality than lower osmolality groups at discharge (Table 2), which showed those who had extremely elevated osmolality in the acute phase may suffer from some unfavorable factors which permanently raise the plasma osmolality. It is possible that those who have higher osmolality both on admission and at discharge are exposed to excessive RAAS activation, and the immediate and sustainable handling of this overactivation should be considered. Although various RAAS blockers have shown definite clinical benefits in HFrEF patients, including angiotensin converting enzyme inhibitors [35], angiotensin II receptor blockers [36], angiotensin-neprilysin inhibitors [37], and mineral corticoid-receptor antagonists [38], the benefits in HFpEF patients are controversial [39]. We propose that further investigation to determine whether these approaches are particularly favorable to HFpEF patients with higher plasma osmolality is warranted.

### Limitations

Several limitations of this study should be mentioned. First, we diagnosed included patients as HFpEF based on the presence of symptom and/or signs of HF, LVEF measurement, and elevated natriuretic peptides. There were lacking for the key structural alterations such as left atrial volume index, left ventricular mass index and E/e′ elevation and stress test assessment, which are proposed to be necessary for HFpEF diagnosis in the 2016 ESC guidelines [1] Second, although our results showed that elevated plasma osmolality on admission was associated with poor outcomes, we could not examine whether extremely decreased plasma osmolality affected prognosis because only 42 (4.4%) subjects had < 275 mOsm/kg on admission. Of note, this finding that excessively low plasma osmolality may be rare in acute decompensated HFpEF patients is important. Third, there have been several formulas which are able to calculate plasma osmolality, and Fazekas et al. reported a formula developed by Zander showed excellent concordance with measured osmolality [40]. Zander’s formula included lactate and bicarbonate to calculate osmolality, however, we have not measured these parameters in our study. We selected the formula consisted of sodium, blood urea nitrogen and glucose, which was also used in the previous article investigated among HFrEF patients [14]. Fourth, plasma osmolality on admission was measured in the period between admission and approximately 48 h after admission. We were not able to assure whether the osmolality was measured prior to any initial treatments including loop diuretics administration and to avoid those initial treatment effects. Fifth, the present study was a multicenter prospective Asian cohort with quite elder patients (median age of as high as 83 years), which would limit the generalizability of the current findings for other races. Sixth, despite multivariable analysis, residual confounding from unmeasured factors may have affected the results. Finally, although we speculated that RAAS activity, and not AVP activity, was responsible for the poor outcomes, we did not measure either urine osmolality or neurohormonal factors substituting for RAAS.

## Conclusion

We show here higher plasma osmolality on admission was associated with the composite endpoint of cardiac mortality or re-admission for HF in HFpEF patients. Further investigation to confirm the results of this small study and to support our understanding of the pathophysiological meaning of plasma osmolality in HFpEF patients is warranted.

## Supplementary Information

**Additional file 1**. **Figure S1**. Correlation of plasma osmolality on admission with each calculation components and eGFR. **Figure S2**. Kaplan-Meier survival curves for composite endpoint, stratified with quantiles of plasma osmolality at discharge. **Appendix S1**. PURSUIT-HFpEF study investigators, institutions.

## Data Availability

Data sharing is not applicable to this article as no datasets were generated or analyzed during the current study.

## Abbreviations

ADHF: Acute decompensated heart failure
AVP: Arginine vasopressin
BNP: Brain natriuretic peptide
CIs: Confidence intervals
GFR: Glomerular filtration rate
GNRI: Geriatric Nutritional Risk Index
HF: Heart failure
HFpEF: Heart failure with preserved ejection fraction
HFrEF: Heart failure with reduced ejection fraction
HRs: Hazard ratios
LVEF: Left ventricular ejection fraction
NT-proBNP: N-terminal pro-B-type natriuretic peptide
PVS: Plasma volume status
RAAS: Renin-angiotensin-aldosterone system
SD: Standard deviation

## References

[1] Ponikowski P, Voors AA, Anker SD, Bueno H, Cleland JGF, Coats AJS (2016). 2016 ESC Guidelines for the diagnosis and treatment of acute and chronic heart failure: The Task Force for the diagnosis and treatment of acute and chronic heart failure of the European Society of Cardiology (ESC) Developed with the special contribution of the Heart Failure Association (HFA) of the ESC. Eur Heart J.

[2] Redfield MM (2016). Heart failure with preserved ejection fraction. N Engl J Med.

[3] Borlaug BA (2014). The pathophysiology of heart failure with preserved ejection fraction. Nat Rev Cardiol.

[4] Rasouli M (2016). Basic concepts and practical equations on osmolality: biochemical approach. Clin Biochem.

[5] Gheorghiade M, Abraham WT, Albert NM, Gattis Stough W, Greenberg BH, O'Connor CM (2007). Relationship between admission serum sodium concentration and clinical outcomes in patients hospitalized for heart failure: an analysis from the OPTIMIZE-HF registry. Eur Heart J.

[6] Jujo K, Minami Y, Haruki S, Matsue Y, Shimazaki K, Kadowaki H (2017). Persistent high blood urea nitrogen level is associated with increased risk of cardiovascular events in patients with acute heart failure. ESC Heart Failure.

[7] Mebazaa A, Gayat E, Lassus J, Meas T, Mueller C, Maggioni A (2013). Association between elevated blood glucose and outcome in acute heart failure: results from an international observational cohort. J Am Coll Cardiol.

[8] Prenner SB, Pillutla R, Yenigalla S, Gaddam S, Lee J, Obeid MJ (2020). Serum albumin is a marker of myocardial fibrosis, adverse pulsatile aortic hemodynamics, and prognosis in heart failure with preserved ejection fraction. J Am Heart Assoc.

[9] Smith GL, Lichtman JH, Bracken MB, Shlipak MG, Phillips CO, DiCapua P (2006). Renal impairment and outcomes in heart failure: systematic review and meta-analysis. J Am Coll Cardiol.

[10] Chatterjee K (2005). Neurohormonal activation in congestive heart failure and the role of vasopressin. Am J Cardiol.

[11] Robertson GL, Shelton RL, Athar S (1976). The osmoregulation of vasopressin. Kidney Int.

[12] Riegger AJ, Liebau G (1982). The renin-angiotensin-aldosterone system, antidiuretic hormone and sympathetic nerve activity in an experimental model of congestive heart failure in the dog. Clin Sci (London, England 1979)..

[13] Vaduganathan M, Marti CN, Mentz RJ, Greene SJ, Ambrosy AP, Subacius HP (2016). Serum osmolality and postdischarge outcomes after hospitalization for heart failure. Am J Cardiol.

[14] Kaya H, Yucel O, Ege MR, Zorlu A, Yucel H, Gunes H (2017). Plasma osmolality predicts mortality in patients with heart failure with reduced ejection fraction. Kardiol Pol.

[15] Arevalo-Lorido JC, Gomez JC, Formiga F, Conde-Martel A, Carrera-Izquierdo M, Muela-Molinero A (2016). High serum osmolarity at admission determines a worse outcome in patients with heart failure: Is a new target emerging?. Int J Cardiol.

[16] Suna S, Hikoso S, Yamada T, Uematsu M, Yasumura Y, Nakagawa A (2020). Study protocol for the PURSUIT-HFpEF study: a prospective, multicenter, observational study of patients with heart failure with preserved ejection fraction. BMJ Open.

[17] Matsumura Y, Hattori A, Manabe S, Takahashi D, Yamamoto Y, Murata T (2017). Case report form reporter: a key component for the integration of electronic medical records and the electronic data capture system. Stud Health Technol Inform.

[18] Kinugasa Y, Kato M, Sugihara S, Hirai M, Yamada K, Yanagihara K (2013). Geriatric nutritional risk index predicts functional dependency and mortality in patients with heart failure with preserved ejection fraction. Circ J.

[19] Ling HZ, Flint J, Damgaard M, Bonfils PK, Cheng AS, Aggarwal S (2015). Calculated plasma volume status and prognosis in chronic heart failure. Eur J Heart Fail.

[20] Lang RM, Badano LP, Mor-Avi V, Afilalo J, Armstrong A, Ernande L (2015). Recommendations for cardiac chamber quantification by echocardiography in adults: an update from the American Society of Echocardiography and the European Association of Cardiovascular Imaging. J Am Soc Echocardiogr.

[21] Fazekas AS, Funk GC, Klobassa DS, Ruther H, Ziegler I, Zander R (2013). Evaluation of 36 formulas for calculating plasma osmolality. Intensive Care Med.

[22] Rusinaru D, Tribouilloy C, Berry C, Richards AM, Whalley GA, Earle N (2012). Relationship of serum sodium concentration to mortality in a wide spectrum of heart failure patients with preserved and with reduced ejection fraction: an individual patient data meta-analysis(dagger): Meta-Analysis Global Group in Chronic heart failure (MAGGIC). Eur J Heart Fail.

[23] Bie P, Damkjaer M (2010). Renin secretion and total body sodium: pathways of integrative control. Clin Exp Pharmacol Physiol.

[24] Oren RM (2005). Hyponatremia in congestive heart failure. Am J Cardiol.

[25] Rowe JW, Minaker KL, Sparrow D, Robertson GL (1982). Age-related failure of volume-pressure-mediated vasopressin release. J Clin Endocrinol Metab.

[26] Boorsma EM, Ter Maaten JM, Damman K, Dinh W, Gustafsson F, Goldsmith S (2020). Congestion in heart failure: a contemporary look at physiology, diagnosis and treatment. Nat Rev Cardiol.

[27] Christ-Crain M, Fenske W (2016). Copeptin in the diagnosis of vasopressin-dependent disorders of fluid homeostasis. Nat Rev Endocrinol.

[28] Schurtz G, Lamblin N, Bauters C, Goldstein P, Lemesle G (2015). Copeptin in acute coronary syndromes and heart failure management: state of the art and future directions. Arch Cardiovasc Dis.

[29] Zhong Y, Wang R, Yan L, Lin M, Liu X, You T (2017). Copeptin in heart failure: review and meta-analysis. Clinica Chimica Acta Int J Clin Chem..

[30] Hage C, Lund LH, Donal E, Daubert JC, Linde C, Mellbin L (2015). Copeptin in patients with heart failure and preserved ejection fraction: a report from the prospective KaRen-study. Open Heart..

[31] Omote K, Nagai T, Kamiya K, Aikawa T, Tsujinaga S, Kato Y (2019). Long-term prognostic significance of admission tricuspid regurgitation pressure gradient in hospitalized patients with heart failure with preserved ejection fraction: a report from the Japanese Real-World Multicenter Registry. J Cardiac Fail.

[32] Palazzuoli A, Ruocco G, Beltrami M, Nuti R, Cleland JG (2018). Combined use of lung ultrasound, B-type natriuretic peptide, and echocardiography for outcome prediction in patients with acute HFrEF and HFpEF. Clin Res Cardiol.

[33] Huang A, Qi X, Hou W, Qi Y, Zhao N, Liu K (2018). Prognostic value of sST2 and NT-proBNP at admission in heart failure with preserved, mid-ranged and reduced ejection fraction. Acta Cardiol.

[34] Carrasco-Sanchez FJ, Galisteo-Almeda L, Paez-Rubio I, Martinez-Marcos FJ, Camacho-Vazquez C, Ruiz-Frutos C (2011). Prognostic value of cystatin C on admission in heart failure with preserved ejection fraction. J Cardiac Fail.

[35] Yusuf S, Pitt B, Davis CE, Hood WB, Cohn JN (1991). Effect of enalapril on survival in patients with reduced left ventricular ejection fractions and congestive heart failure. N Engl J Med.

[36] McMurray JJ, Ostergren J, Swedberg K, Granger CB, Held P, Michelson EL (2003). Effects of candesartan in patients with chronic heart failure and reduced left-ventricular systolic function taking angiotensin-converting-enzyme inhibitors: the CHARM-Added trial. Lancet (London, England).

[37] McMurray JJ, Packer M, Desai AS, Gong J, Lefkowitz MP, Rizkala AR (2014). Angiotensin-neprilysin inhibition versus enalapril in heart failure. N Engl J Med.

[38] Pitt B, Zannad F, Remme WJ, Cody R, Castaigne A, Perez A (1999). The effect of spironolactone on morbidity and mortality in patients with severe heart failure. Randomized Aldactone Evaluation Study Investigators. N Engl J Med.

[39] Solomon SD, McMurray JJV, Anand IS, Ge J, Lam CSP, Maggioni AP (2019). Angiotensin-neprilysin inhibition in heart failure with preserved ejection fraction. N Engl J Med.

[40] Fazekas AS, Funk GC, Klobassa DS, Rüther H, Ziegler I, Zander R (2013). Evaluation of 36 formulas for calculating plasma osmolality. Intensive Care Med.

