# Supplementary material for: Prognostic relevance of elevated plasma osmolality on admission in acute decompensated heart failure with preserved ejection fraction: insights from PURSUIT-HFpEF registry
Source: BMC Cardiovasc Disord. 2021 Jun 7;21:281. doi: 10.1186/s12872-021-02098-z (PMC8182926; doi:10.1186/s12872-021-02098-z)
Supplement: Supplementary file 1 — Additional file 1. Figure S1. Correlation of plasma osmolality on admission with each calculation components and eGFR. Figure S2. Kaplan-Meier survival curves for composite endpoint, stratified with quantiles of plasma osmolality at discharge. Appendix S1. PURSUIT-HFpEF study investigators, institutions. [file 12872_2021_2098_MOESM1_ESM.pdf]

Supplementary materials

Prognostic relevance of elevated plasma osmolality on admission in acute decompensated heart failure with preserved ejection fraction: insights from PURSUIT-HFpEF registry

Akito Nakagawa, Yoshio Yasumura, Chikako Yoshida, Takahiro Okumura, Jun Tateishi, Junichi Yoshida, Shunsuke Tamaki, Masamichi Yano, Takaharu Hayashi, Yusuke Nakagawa, Takahisa Yamada, Daisaku Nakatani, Shungo Hikoso, and Yasushi Sakata on behalf of Osaka CardioVascular Conference (OCVC)-Heart Failure Investigators

Online Figure S1. Correlation of plasma osmolality on admission with each calculation components and eGFR

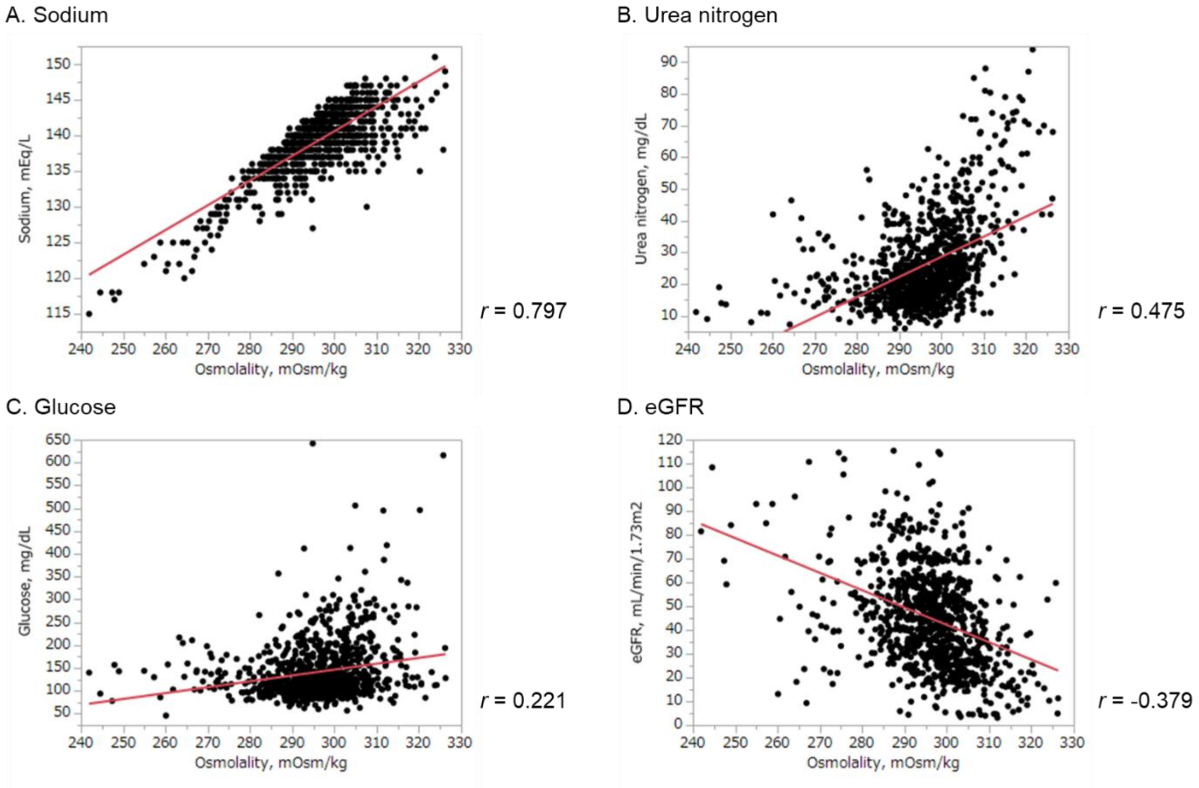

Correlation of plasma osmolality on admission and serum sodium (A), blood urea nitrogen (B), blood glucose (C), and eGFR (D) are shown.

Abbreviations: eGFR, estimated glomerular filtration rate

Online Figure S2. Kaplan-Meier survival curves for composite endpoint, stratified with quantiles of plasma osmolality at discharge

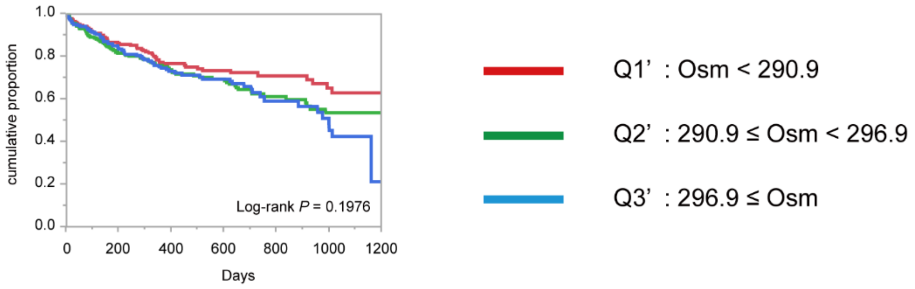

Number at risk

Q1	295	103	43	4
Q2	296	110	45	8
Q3	296	90	29	2

Composite endpoint was defined as cardiac mortality or heart failure re-admission.

Abbreviations: Osm, plasma osmolality (mOsm/kg) at discharge

Appendix S1. PURSUIT-HFpEF study investigators, institutions

The OCVC-Heart Failure Investigators

Chair: Yasushi Sakata, Department of Cardiovascular Medicine, Osaka University Graduate School of Medicine, 2-2 Yamada-oka, Suita 565-0871, Japan

Secretariat: Shungo Hikoso (Chief), Daisaku Nakatani, Hiroya Mizuno, Shinichiro Suna, Katsuki Okada, Tomoharu Dohi, Yohei Sotomi, Takayuki Kojima, Akihiro Sunaga, Hirota Kida, Bolrathanak Oeun, and Taiki Sato; Department of Cardiovascular Medicine, Osaka University Graduate School of Medicine, Suita, Japan.

Investigators:

Shunsuke Tamaki, Tetsuya Watanabe, and Takahisa Yamada, Osaka General Medical Center, Osaka, Japan; Takaharu Hayashi and Yoshiharu Higuchi, Osaka Police Hospital, Osaka, Japan; Masaharu Masuda, Mitsutoshi Asai, and Toshiaki Mano, Kansai Rosai Hospital, Amagasaki, Japan; Hisakazu Fuji, Kobe Ekisaikai Hospital, Kobe, Japan; Daisaku Masuda, Yoshihiro Takeda, Yoshiyuki Nagai, and Shizuya Yamashita, Rinku General Medical Center, Izumisano, Japan; Masami Sairyo, Yusuke Nakagawa and Shuichi Nozaki, Kawanishi City Hospital, Kawanishi, Japan; Haruhiko Abe, Yasunori Ueda, Masaaki Uematsu, and Yukihiro Koretsune, National Hospital Organization Osaka National Hospital, Osaka, Japan; Kunihiko Nagai, Ikeda Municipal Hospital, Ikeda, Japan; Masamichi Yano, Masami Nishino, and Jun Tanouchi, Osaka Rosai Hospital, Sakai, Japan; Yoh Arita and Shinji Hasegawa, Japan Community Health Care Organization Osaka Hospital, Osaka, Japan; Takamaru Ishizu, Minoru Ichikawa and Yuzuru Takano, Higashiosaka City Medical Center, Higashiosaka, Japan; Eisai Rin, Kawachi General Hospital, Higashiosaka, Japan; Yukinori Shinoda and Shiro Hoshida, Yao Municipal Hospital, Yao, Japan; Masahiro Izumi, Kinki Central Hospital, Itami, Japan; Hiroyoshi Yamamoto and Hiroyasu Kato, Japan Community Health Care Organization, Osaka Minato Central Hospital, Osaka, Japan; Kazuhiro Nakatani and Yuji Yasuga, Sumitomo Hospital, Osaka, Japan; Mayu Nishio and Keiji Hirooka, Saiseikai Senri Hospital, Suita, Japan; Takahiro Yoshimura and Yoshinori Yasuoka, National Hospital Organization Osaka Minami Medical Center, Kawachinagano, Japan; Akihiro Tani, Kano General Hospital, Osaka, Japan; Yasushi Okumoto and Hideharu Akagi, Kinan

Hospital, Tanabe, Japan; Yasunaka Makino, Hyogo Prefectural Nishinomiya Hospital, Nishinomiya, Japan; Toshinari Onishi and Katsuomi Iwakura, Sakurabashi Watanabe Hospital, Osaka, Japan; Nagahiro Nishikawa and Yoshiyuki Kijima, Japan Community Health Care Organization, Hoshigaoka Medical Center, Hirakata, Japan; Takashi Kitao and Hideyuki Kanai, Minoh City Hospital, Minoh, Japan; Wataru Shioyama and Masashi Fujita, Osaka International Cancer Institute, Osaka, Japan; Koichiro Harada, Suita Municipal Hospital, Suita, Japan; Masahiro Kumada and Osamu Nakagawa, Toyonaka Municipal Hospital, Toyonaka, Japan; Ryo Araki and Takayuki Yamada, Otemae Hospital, Osaka, Japan; Akito Nakagawa and Yoshio Yasumura, Amagasaki Chuo Hospital, Amagasaki, Japan; and Taiki Sato, Akihiro Sunaga, Bolrathanak Oeun, Hirota Kida, Takayuki Kojima, Yohei Sotomi, Tomoharu Dohi, Kei Nakamoto, Katsuki Okada, Fusako Sera, Shinichiro Suna, Hidetaka Kioka, Tomohito Ohtani, Toshihiro Takeda, Daisaku Nakatani, Hiroya Mizuno, Shungo Hikoso, Yasushi Matsumura and Yasushi Sakata, Osaka University Graduate School of Medicine, Suita, Japan.
